# Assessment of the efficiency of synergistic photocatalysis on penicillin G biodegradation by whole cell Paracoccus sp

**DOI:** 10.1186/s13036-021-00275-4

**Published:** 2021-10-27

**Authors:** Peng Wang, Shouxin Liu, Xiaochun Wang, Qinqin Cong, Jialin Lu

**Affiliations:** 1 College of Chemical & Pharmaceutical Engineering, Hebei University of Science & Technology, Shijiazhuang, 050018 China; 2 State Key Laboratory Breeding Base-Hebei Province Key Laboratory of Molecular Chemistry for Drug, Hebei University of Science & Technology, Shijiazhuang, 050018 China; 3 Hebei Province Pharmaceutical Chemical Engineering Technology Research Center, Shijiazhuang, 050018 China

**Keywords:** Synergistic catalysis, Visible light, Biodegradation, Penicillin G, *Paracoccus sp.* strain

## Abstract

**Background:**

The *Paracoccus* sp. strain isolated from sludge was identified and evaluated for catalytic activity in the degradation of penicillin G.

**Results:**

High degradation efficiency and synergistic catalytic effects of the whole cell and visible light without additional catalysts were observed. The key factors influencing the degradation and kinetics of penicillin G were investigated. The results showed the phenylacetic acid, which was produced during penicillin G biodegradation, exhibited stronger inhibiting effects on KDSPL-02. However, this effect was reduced by visible light irradiation without any additional photocatalyst; furthermore, the rate of penicillin G biodegradation was accelerated, reaching a 100% rate in 12 h at a penicillin G concentration of 1.2 g/L. Four key intermediates produced during penicillin G degradation were isolated and identified by LC–MS, ^1^H NMR, and ^13^C NMR. Enzymes involved in the PAA pathway were proposed from a genomic analysis of KDSPL-02.

**Conclusions:**

These results provide a new method for bio-degrading of penicillin or other antibiotic pollutants using photoaccelerating biocatalysts with greater efficiency and more environmentally friendly conditions.

**Supplementary Information:**

The online version contains supplementary material available at 10.1186/s13036-021-00275-4.

## Introduction

In recent years, the irrational use of antibiotics has led to worldwide environmental pollution attributed to clinical prescriptions, animal husbandry abuse and fermentation fungi residue [1–3]. Among various antibiotics, penicillin G is popular and widely used in humans and animals [4–7]. Penicillin G has the highest activity among natural antibiotics and more than 11,000 tons are produced annually in 20 countries [8, 9]. However, with increased use, penicillin G contamination has been widely detected in surface water, groundwater, sewage water, and sometimes in drinking water [10, 11]. Penicillin G and its intermediates could cause potential secondary water pollution [12, 13]. Penicillin is unstable in the environment; its degradation intermediate metabolite penicilloic acid can cause allergic reactions [14]. Other degradation products, such as amino-penicillanic acid, reversibly synthesizes penicillin and other stable β-lactams [15–17]. Furthermore, a thiazole intermediate degradation product has potential environmental pollution risks [18]. Thus, the full mineralized degradation of penicillin into inorganic substances is of great significance [19, 20].

With respect to biodegradation mechanisms, most β-lactam antibiotic degradation studies have focused on the separation and characterization of key intermediate metabolites and not the whole pathway, including the mineralization products [21–24]. HPLC-mass spectrometry has typically been used as the primary detection method; however, such a tool cannot absolutely confirm the structure of degradation products or fully clarify the changes in the degradation processes [25]. A characterization of the structure of intermediates will assist in characterizing and developing functional degradation enzymes. Therefore, more accurate structural analyses are necessary, such as ^1^H NMR and ^13^C NMR spectroscopy, to confirm the structure of degradation products in the biodegradation of antibiotics.

Regarding the technology for β-lactam antibiotic degradation, various methods such as nanofiltration [26], ozonation [27, 28], biodegradation [21], surface adsorption [29], advanced oxidation [19, 30] and photocatalytic processes [22, 31] have been reported to remove β-lactam antibiotics. However, biodegradation typically results in incomplete mineralization; furthermore, low degradation efficiency has been observed by several authors [32, 33]. Mineralization degradation of antibiotic residues occur at different scales, including in wastewater, antibiotic mycelium, and other ecological environments.

The combination of biodegradation and photocatalysis is a promising technology that can achieve the complete mineralization of contaminants [34–37]. The process is dependent on typically independent biocatalytic and photocatalytic reactions. Although the coupling of these two processes has been studied for antibiotic degradation, almost all reported methods have required additive materials or fibrous structures, which are economically prohibitive and practically difficult to achieve [37]. The current research on combined photocatalysis and biodegradation is relatively new and there are still many unresolved issues in its practical applications, such as the high costs of photocatalysts and carriers and light reflection and occlusion in wastewaters. One of the most promising ways to overcome these problems is to simplify the photo-biodegradation system by focusing on biocatalysts rather than photocatalysts.

In this work, we used penicillin G as a model contaminant for the biocatalytic mineralized degradation of penicillin G under visible light irradiation without any photocatalyst. Whole *Paracoccus sp*. strain cells isolated from sludge contaminated by antibiotics were utilized as biocatalysts; this strain has been shown to solely use penicillin G as an energy and carbon source. The effect of heterogeneous photocatalysis on the improvement in biodegradation efficiency of penicillin G was evaluated. Penicillin G degradation products were assessed and identified using HPLC, LC–MS, ^1^H NMR, and ^13^C NMR. This study describes an efficient photo-assisted biodegradation system that can be used for the complete mineralization and degradation of penicillin in the environment.

## Materials and methods

### Materials

Sludge samples were collected from the district of Shijiazhuang, China. Penicillin G was provided by North China Pharmaceutical Group Corporation, China. All other chemicals used in this study were analytical grade and commercially available without further purification unless otherwise noted. Wastewater contained penicillin G was derived from North China Pharmaceutical Group Corporation.

### Culture medium

The base mineral media (BMM) consisted of 1.60 g of K_2_HPO_4_, 0.40 g of KH_2_PO_4_, 0.20 g of MgSO_4_·7H_2_O, 0.03 g of CaCl_2_·2H_2_O, 0.02 g of FeCl_3_·6H_2_O, 0.50 g of NH_4_NO_3_, and 0.50 g of yeast extract per litre of water.

The growth medium (GM) consisted of 2.40 g of yeast extract per litre of water in the base of BMM medium. The final pH was adjusted to 7.0.

The fermentation medium (FM) was composed of the following: 10 mL of industrial liquid sugar; 2.40 g of yeast extract, 1.60 g of K_2_HPO_4_, 0.40 g of KH_2_PO_4_, 0.20 g of MgSO_4_•7H_2_O, 0.03 g of CaCl_2_•2H_2_O, 0.02 g of FeCl_3_•6H_2_O, 0.50 g of NH_4_NO_3_ per litre of water; and 45 mg L^− 1^ penicillin G as the inducer.

### *Paracoccus sp.* KDSPL-02 cultivation using penicillin as the sole carbon and energy source

To test whether strain KDSPL-02 can using penicillin as the sole carbon and energy source, varies carbon sources such as glucose (1.0 g/L), sucrose (1.0 g/L), maltose (1.0 g/L) and starch (1.0 g/L), were tested in addition to penicillin (0.1 g/L) in BMM. As a control, the base mineral media contained no carbon sources and comprised NH_4_NO_3_ (1 g/L), NaCl (0.5 g/L), K_2_HPO_4_ (0.5 g/L), MgSO_4_ (0.5 g/L), and FeSO_4_ (0.01 g/L).

### Optimization *of Paracoccus sp.* KDSPL-02 whole cell fermentation

To accumulate high titres for the biodegradation of penicillin G. the fermentation of strain KDSPL-02 was optimized. Inoculation medium was prepared with 0.01 mg of nickel chloride hexahydrate, 0.02 mg of sodium molybdate dihydrate, 0.01 mg of cobalt chloride, 0.1 mg of zinc sulphate heptahydrate, 0.005 mg of riboflavin, and 0.01 mg of pyridoxal hydrochloride per litre of water. The pH before autoclaving was within 6.8–7.0 (adjusted with 12% ammonia solution). Flasks were inoculated with the bacteria and placed on an orbital shaker. The agitation velocity was 200 rpm, and the volume of the flask was 250 mL. The activity (U) of penicillin G degraded by whole KDSPL-02 cells was defined as g/g·h, i.e., the wet cell of one gram degrading penicillin (g) over 1 hr. All experiments were conducted in triplicate biodegradation reactors and triplicate no-cell control reactors in dark to prevent photodegradation.

### Penicillin G degradation by whole KDSPL-02 cells under visible light irradiation

A suspension solution (0.2 mL) of whole KDSPL-02 cells with a degradation activity of 2.0–7.0 U was added to a 250-mL conical flask containing a 100-mL solution of 0.8–1.6 g L^− 1^ penicillin G. The treatment was carried out at 120 rpm at 32 °C under visible light irradiation. Initially, persistent organic compounds were converted into more easily photodegradable compounds, thereby promoting photodegradation. The photocatalytic degradation of penicillin G was carried out in an annular reactor under visible light or LED irradiation with a medium mercury lamp (TQ 718 Z1 700 W) purchased from Heraeus at the reactor centre. Irradiation below 290 nm was filtered by a Duran cooling tube surrounding the lamp. The reaction was monitored via HPLC at 1 h intervals. Degradation was completed in approximately 12–14 h. Degradation intermediates were separated by HPLC.

### Phenylacetic acid inhibition test

To verify whether phenylacetic acid has an inhibitory effect on KSDPL-02, we conducted a phenylacetic acid inhibition test. Various concentrations of phenylacetic acid (0.01 g/L, 0.05 g/L, 0.1 g/L, and 0.2 g/L) were added to culture medium, and changes in OD600 were used to characterize the inhibition of phenylacetic acid.

Furthermore, the degradation of phenylacetic acid by KDSPL-02 under light and non-light conditions was investigated. Culture medium was modified with 0.1 g/L of phenylacetic acid to investigate the clearance rate of phenylacetic acid at different times (0, 2, 4, 6, 8, 10, and 12 h) under light and non-light conditions.

### Analytic methods

The light absorption properties were measured using UV–vis diffuse reflectance spectrophotometer (DRS, JASCO, UV-550) with a wavelength range of 200–900 nm. A 300 W xenon lamp (λ > 290 nm, PLS-SXE300CUV, Perfectlight Instruments Co. Ltd., Beijing) was used as light source, and the average light intensity was 78.5mw/cm − 2 (UV-A radiation meter). LC-MS data were measured with Thermo Scientific ISQ QD. ^1^HNMR and ^13^CNMR were recorded on a Bruker Avance II 500 spectrometer in CDCl_3_ unless stated otherwise, using tetramethylsilane as an internal reference, operated at 500.13 MHz for ^1^H, and 125 MHz for ^13^C. J values are given in Hz. Penicillin and intermediate concentrations were measured via HPLC (Dionex and Agilent) with an UltimateR XB C18 column (4.6 × 250 mm, 5 μm) at a flow rate of 1.0 mL min-1, column temperature of 30 °C, mobile phase of methanol and phosphate (0.1 M potassium dihydrogen phosphate, pH 3.5) at a ratio of 50:50, injection volume of 20 μL, and detection wavelength of 225 nm.

## Results and discussion

### *Paracoccus sp.* KDSPL-02 can use penicillin G as the sole carbon and energy source

**Fig. 1:**
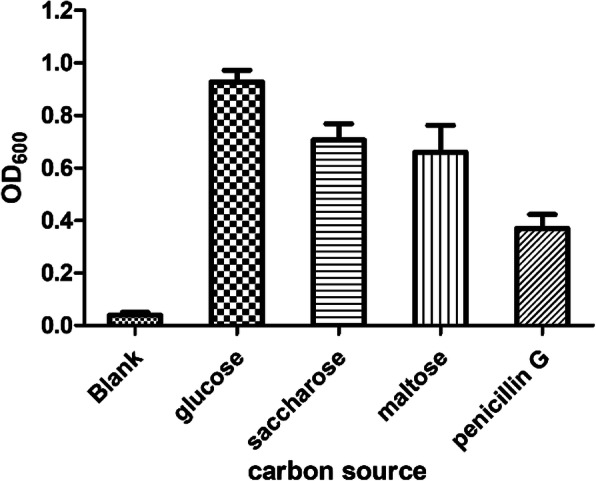
the growth of KDSPL-02 when using varies substance (glucose, sucrose, starch or penicillin G) as the sole carbon and energy source

First, the growth of KDSPL-02 when using penicillin as the sole carbon and energy source was investigated. As shown in Fig. 1, when using glucose, sucrose or starch as the single carbon source, the growth of KDSPL-02 was better, and the OD_600_ after 6 h of growth reached 1.2, 0.9 and 0.9, respectively. When penicillin was used as the sole carbon and energy source, the growth of KDSPL-02 was relatively poor with the OD_600_ reaching 0.4 after 6 h of growth. Although the growth status of KDSPL-02 was poor when using penicillin, this showed that KDSPL-02 could still survive.

Most microorganisms are not known to use antibiotics as sole carbon and energy sources; co-metabolism is often required to maintain growth [38]. In our study, the strain KDSPL-02 could advantageously use penicillin as the sole carbon and energy source. This ability is necessary for degrading antibiotics. The biomineralization of an antibiotic is the complete metabolism of the antibiotic, i.e., bacterial cells first decompose the antibiotic into small molecular intermediate degradation products that can be used by the bacteria through specific degradative enzymes. These intermediate degradation products then pass the central bacterial metabolic pathway for breakdown into carbon dioxide and water to realize the complete degradation of antibiotics.

### Optimization of penicillin biotransformation by *Paracoccus sp*. KDSPL-02

Because penicillin hydrolyses in water, penicillin biotransformation in solution cannot be directly measured. Hence, all experiments included control treatment reactors that contained no cells. Only hydrolysis occurred in the control reactors, whereas both hydrolysis and biotransformation occurred in the bacterial treatment reactors. Observed differences in penicillin concentrations with the control reactors were then attributed to biotransformation.

#### Effects of *Paracoccus sp*. KDSPL-02 cell dry weight on penicillin biotransformation

**Fig. 2:**
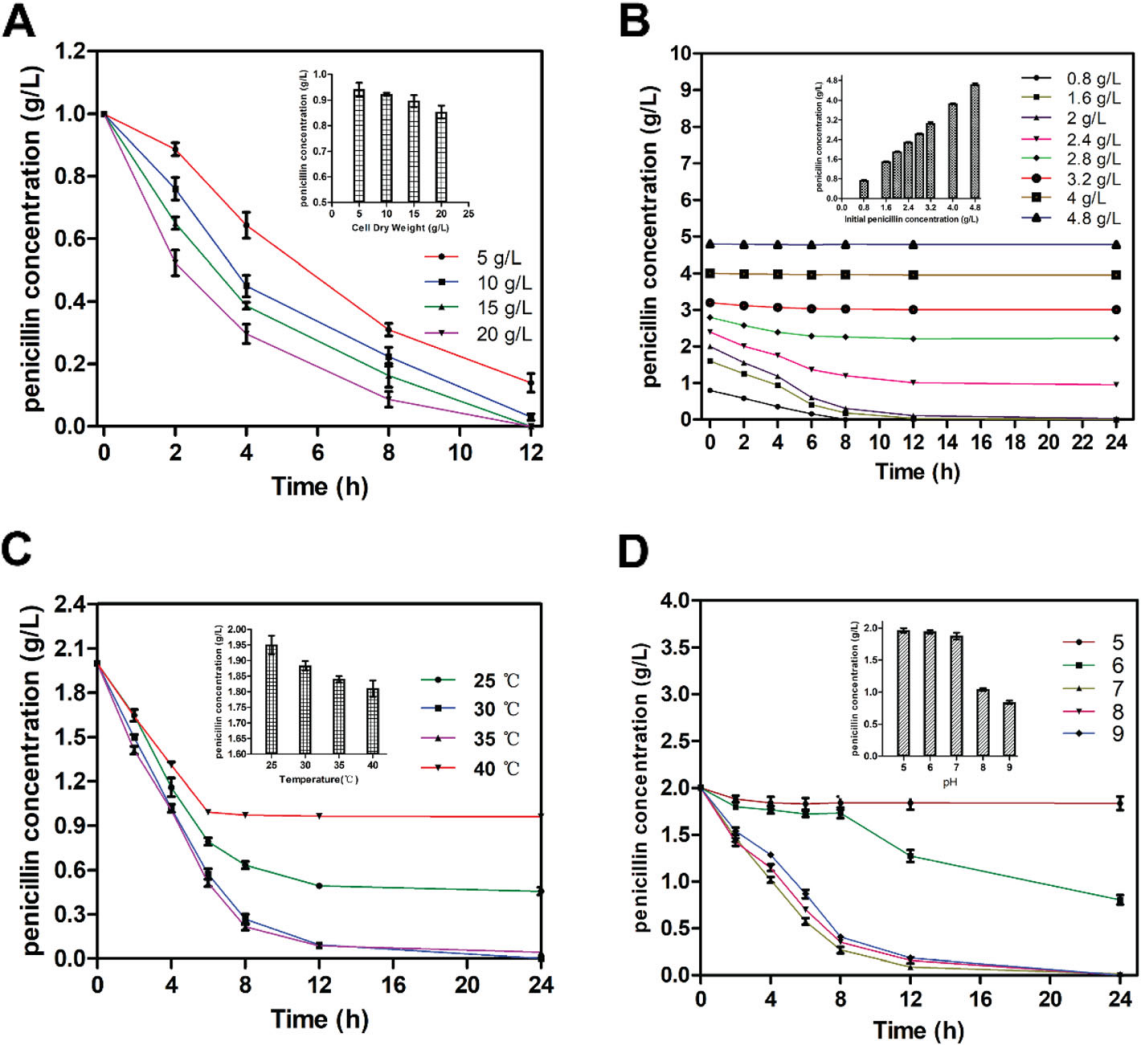
Effect of cell dry weight of KDSPL-02 whole cell (**a**), initial penicillin G concentration (**b**), temperature (**c**), and pH (**d**) on degradation rate by *Paracoccus sp*. KDSPL-02

The biodegradation conditions were optimized by single-factor tests under different conditions, such as *Paracoccus sp*. KDSPL-02 cell dry weight (5 g/L- 20 g/L). The research result showed that the *Paracoccus sp.* KDSPL-02 cell dry weight was related to the degradation efficiency. The degradation rate increased steadily with increasing cell dry weight. When the cell activity was above 10 g/L, the degradation rate reached to 100% within 12 h (Fig. 2a).

#### Effects of the concentration of the substrate on penicillin biotransformation

The degradation also depended strongly on the concentration of the substrate. As the initial concentration of penicillin G increased (from 0.4 g L^− 1^ to 5.2 g L^− 1^), the degradation ability of the strain lost gradually (Fig. 2b). The degradation rate of penicillin G was approximately 100% in the beginning, and then decreased as the concentration of penicillin G increased, becoming inhibited above 2.0 g L^− 1^. The initial highest concentration of penicillin G optimized was 0.8–1.6 g L^− 1^.

Earlier reports on penicillin G biodegradation by different bacterial strains are summarized in Table S1. The 100% degradation rate presented in this study was comparable to or higher than those in previous studies. For instance, *Phanerochate chrysosporium* tolerates high penicillin G concentrations up to 2000 mg L^− 1^ but can only achieve a degradation efficiency of 61.4% [39]. Furthermore, the penicillin G degradation efficiencies of *Klebsiella pneumoniae* Z-1, *Actinobacillus pleuropneumoniae*, and *Achromobacter* sp. CCM 2428 reach 99.9% or nearly 100% at penicillin G concentrations below 300 and 500 mg L^− 1^, respectively, but an increase in tolerance limits the degradation ability [40–42]. Compared with the above bacterial strains, KDSPL-02 has significant advantages. The free cells completely eliminated penicillin G when its initial concentration was 1.2 g L^− 1^; the cells retained their degradation efficiency to 2.0 g L^− 1^, and the final degradation product was benzoic acid.

#### Effects of temperature and pH on penicillin biotransformation

Biodegradation conditions were optimized by single-factor tests at a temperature range from 25 °C to 40 °C and a pH range from 4.0 to 10.0. To eliminate interference from penicillin hydrolysis on the detection of penicillin biodegradation, we investigated the hydrolysis of penicillin under different conditions.

The temperature and pH of the degradation reaction are two important factors. The degradation rate steadily increased as the temperature increased from 25 °C to 30 °C. When the temperature was raised to 40 °C, degradation efficiency declined. At 1.2 g L^− 1^, penicillin G was almost completely degraded by whole-cell catalysis from 30 °C–35 °C (Fig. 2c).

Because pH changes with degradation, the initial pH of the degradation solution containing penicillin strongly impacted the reaction rate. The effect of the initial pH is plotted in Fig. 2d. The data shows that the degradation rate was lowest at pH 5.0, which indicated that the KDSPL-02 cell enzymes did not function well under acidic conditions. The degradation activity was higher at pH 9.0, indicating that KDSPL-02 whole-cell catalytic degradation of penicillin G occurred under neutral or weakly alkaline conditions, as confirmed in previous studies (Anwar et al., 2009). The highest degradation rate was achieved from pH 7.0–9.0, which indicated that KDSPL-02 was functional over a wide pH range. Additionally, we found that penicillin was unstable in aqueous alkaline solution (when the pH is greater than 8.0) and obvious hydrolysis will occur. Hydrolysis was not observed in acidic (pH 4.0–7.0), neutral (pH 7.0) and weakly alkaline (pH 7.0–8.0) environments.

### Pathway analysis of penicillin G biodegradation by *Parococcus sp.* KDSPL-02

**Fig. 3:**
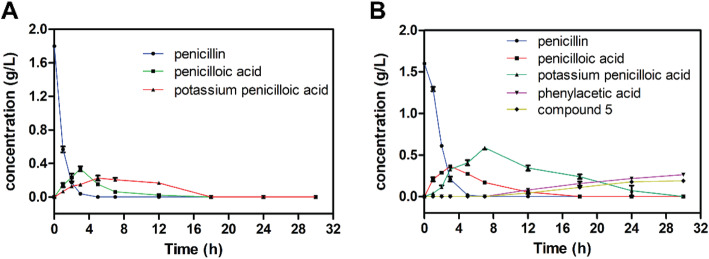
Conversion of penicillin G biodegradation at initial penicillin G concentrations of 0.8 g L^− 1^ (**a**) and 1.6 g L^− 1^ (**b**)

The process of penicillin G degradation via biocatalysis is generally very complex, a few intermediates were shown by HPLC when penicillin G was treated with whole cell KDSPL-02 (Fig. S1). Penicillin G in the medium solution without whole cell KDSPL-02 was not shown obvious degradation detected by HPLC. In addition, the number of intermediates and the values varied with penicillin G concentration. Three peaks appeared in the HPLC when the concentration was below 0.8 g L^− 1^, and the change of each component with the reaction carrying out shown in Fig. 3a. While the concentration of penicillin G was at 1.6 g L^− 1^, five components exhibited in the HPLC, and the conversions were shown in Fig. 3b.

All intermediates attributed to peaks in HPLC were isolated and collected to yield purified samples. The structures of the compounds were determined via ^1^H NMR, ^13^C NMR, and LC–MS. The intermediate compounds corresponding to peak 2 and 3 had the same mass spectrum, the molecular ion peak [M + 1]^**+**^ (*m/z*) was 353.2, and no fragment signal peaks showed a difference in LC–MS (Fig. S2a). Moreover, their ^1^H NMR and ^13^C NMR spectra were highly similar, belonging to potassium 2-(carboxy(2-phenylacetamido)methyl)-5,5-dimethylthiazolidine-4-carboxylate (penicilloic acid)(**2**) and potassium 2-(4-carboxy-5,5-dimethylthiazolidin-2-yl)-2-(2-phenylacetamido) acetate (potassium penicilloic acid)(**3**) (Fig. S3a-b). The former is the product formed by opening the ring of β-lactam in penicillin G, while the latter is its isomer that was formed through the exchange of an H^+^ with potassium ion. The compound corresponding to peak 4 is simple. The LC–MS showed that the molecular ion peak [M + 1]^**+**^ (*m/z*) was 137.3 (Fig. S2b), and ^1^H NMR and ^13^C NMR indicated the presence of a benzene ring in its structure. Thus, this peak was belonged to phenylacetic acid (**4**) (Fig. S4a-b). For the species corresponding to peak No. 5, LC–MS showed a molecular ion peak [M + 1]^**+**^ of 235.1 (Fig. S2c) [13]. Moreover, the characteristic structure of 5,5-dimethylthiazolidine contained in penicillin G was still present in its ^1^H NMR and ^13^C NMR spectra (Fig. S4a-b). Thus, this peak in HPLC belonged to the potassium salt of 2-(amino(carboxyl)methyl)-5,5-dimethylthiazolidine-4-carboxylic acid (**5**) and potassium 2-amino-2-(4-carboxy-5,5-dimethylthiazolindin-2-yl) acetate (**6**).

**Fig. 4:**
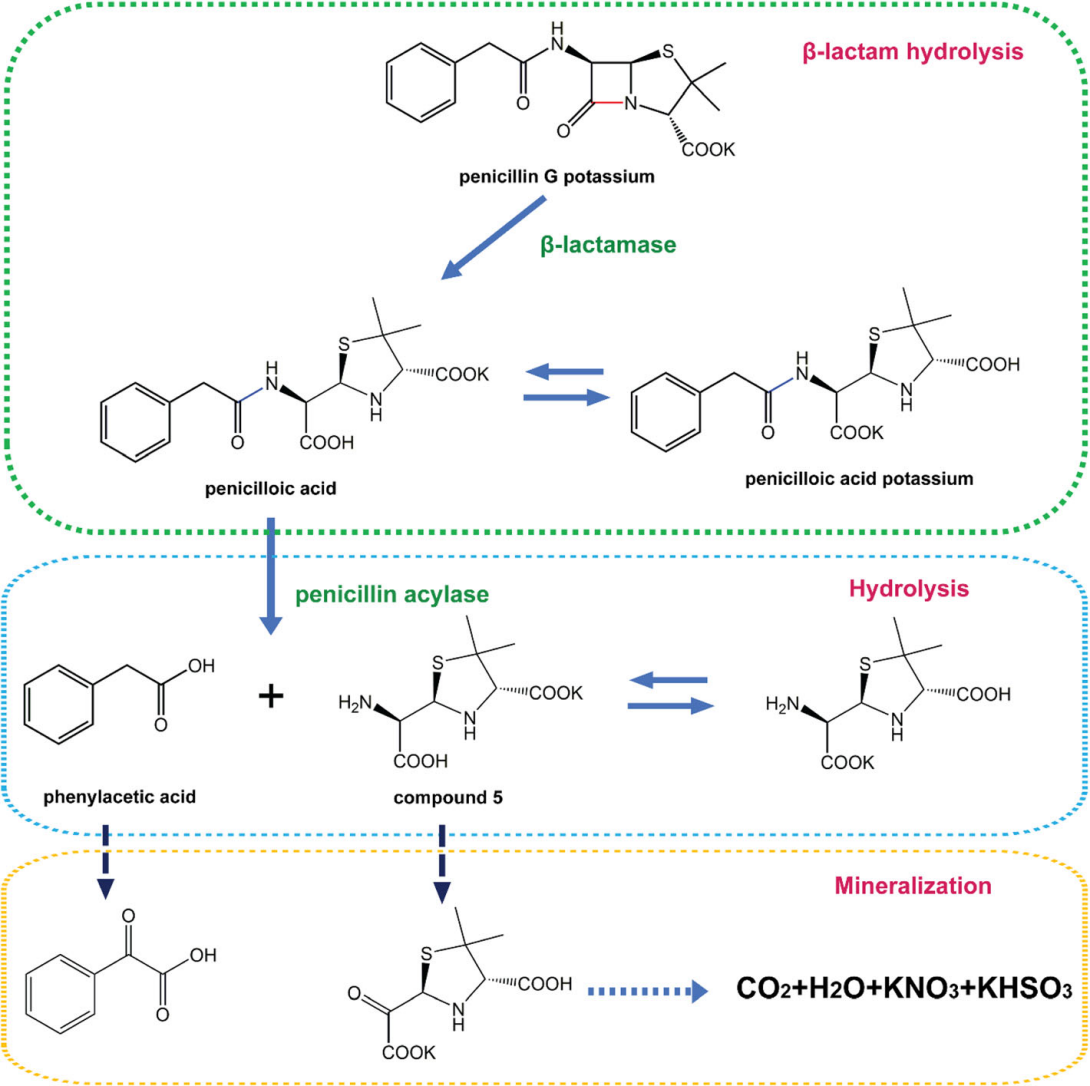
Proposed penicillin G biodegradation pathway by *Paracoccus sp.* KDSPL-02

According to the HPLC, MS and HMR analysis of the intermediates, the possible pathway of complete degradation process catalyzed by KDSPL-02 whole cells was proposed, as shown in Fig. 4. β-Lactam hydrolase initially catalyzed penicillin G to open the β-lactam ring, forming intermediate penicilloic acid, which was simultaneously and reversibly converted with intermediate potassium penicilloic acid. Afterward, the open ring compounds were hydrolyzed by amide hydrolase to form phenylacetic acid and compound **5** and compound **6**. Phenylacetic acid was oxidized by oxidases in the cell through oxophenylacetic acid **7** which coincide with the peak 150.1 in the MS of penicilloic acid degradation mixture to form inorganic mineralizers. Compound **5**, which coincide with the peak 271.1 in the MS of its degradation mixture, was initially converted to the corresponding α-keto acid **8**, which then underwent further decarboxylation, oxidation, and hydrolysis to yield small inorganic compounds.

### Light enhancing the penicillin biodegradation efficiency of *Paracoccus sp.* KDSPL-02

KDSPL-02 was a more effective catalyst of penicillin G degradation under the optimized conditions. Using the same methods for the treatment of penicillin G in solution, wastewater contained penicillin G was treated. The results showed that 0.8–1.6 g L^− 1^ penicillin G was almost degraded completely by whole-cell catalysis at 30 °C. However, in the degradation of penicillin G, in solution or wastewater, the overall reaction rate was subject to secondary reactions. To improve the biodegradation efficiency, visible light irradiation was used to enhance the biocatalytic degradation.

**Fig. 5:**
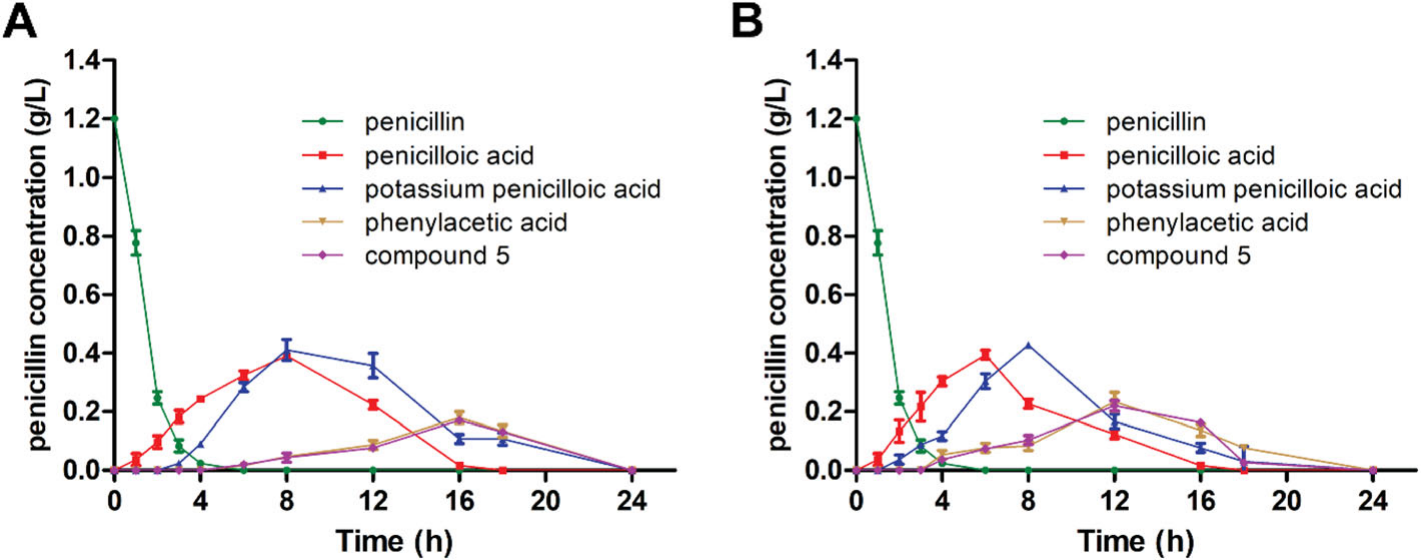
Conversion of penicillin G biodegradation at an initial penicillin G concentration of 1.2 g L^− 1^ (**a**) and using co-catalysis of KDSPL-02 whole cell and light degradation at an initial penicillin G concentration of 1.2 g L^− 1^ (**b**)

Changes in species concentrations with treatment time during the degradation of penicillin G with an initial concentration of 1.2 g L^− 1^ are shown by HPLC data in Fig. 5a. Penicillin G concentration decreased rapidly with degradation reaction. Peak No. 1 (penicillin G) completely disappeared after 5 h at an initial concentration below 1.2 g L^− 1^. However, the conversion rate of penicilloic acid intermediate was slow and required 24 h. Phenylacetic acid and compound **5** intermediates degraded more rapidly than penicilloic acid within 18 h. In the degradation process, the rate-limiting reaction was the conversion of penicilloic acid into phenylacetic acid and compound **5**. To improve the degradation kinetics, we tested auxiliary strategies. The results indicated that visible light markedly accelerated the rate-limiting reaction. Based on HPLC data (Fig. 5b), the degradation time decreased from 24 h to 18 h. The effect of photocatalysis on penicillin G degradation was further studied. The data shown in Table 1 indicates that synergistic catalysis can accelerate the rate of the treatment, i.e., the conversion of penicilloic acid or the conversion of phenylacetic acid and compound **5** into small inorganic molecules. Regarding the heating effect of visible light, the same results were obtained when homogeneous LED lights, such as green and blue, were substituted for visible light. Therefore, the heating effect was not a major factor in the degradation process.

**Table 1:** Effect of co-catalysis of KDSPL-02 whole cell and light degradation

	Concentration (g/L)	Temperature (°C)	Degradation time(h)	Degradation rate (%)
Blank	0.8	31	18	100
	1.2	32	24	99.9
Xenon light	0.8	30	13	100
	1.2	32	18	99.9
Green light	0.8	31	12	100
	1.2	33	18	100
Blue light	0.8	31	13	100
	1.2	32	18	99.8

Previous studies reported penicillin degradation by soil microorganism which shown that penicillin was first catalyzed by beta-lactamase to form penicilloic and then hydrolyzed by penicillin acylase. In this study, the degradation pathway of penicillin was similar with the degradation pathway reported in reference [38]. Furthermore, our previous report exhibited the penicillin V degradation by *Ochrobactrum tritici* X-2. In similar with the degradation pathway here, penicillin V was first degraded by beta-lactamase and then degraded by penicillin acylase [43]. In most recently, several research using β-lactamase to realize penicillin biodegradation. Although it is exhibited high efficiency, the degradation pathway is relatively simple, and only achieve the hydroxylation of penicillin without further mineralization degradation [44, 45]. Our present work was using light irradiation to stimulate the catalytic efficiency of whole cell of KDSPL-02 and also our work realized the mineralization degradation of penicillin.

### Phenylacetic acid removal during visible light treatment

**Fig. 6:**
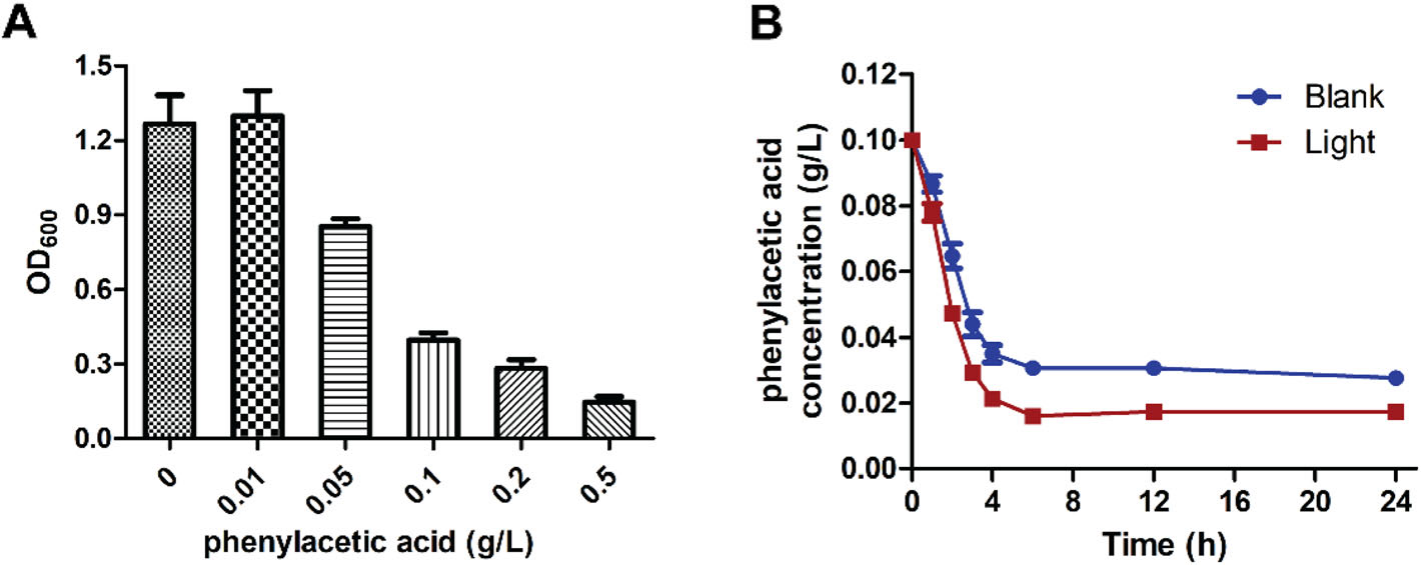
Effect of visible light inducing catalysis on phenylacetic acid oxidation

Phenylacetic acid intermediate often accumulates, especially when treating high concentrations of penicillin G, and exhibits a strong inhibitory effect that could inhibit the growth of the strain as shown in Fig. 6a. Although the function of visible light in co-catalysis was not clearly elucidated, the mechanism by which it induces oxidation was determined. Thus, we speculated that visible light enhances the oxidation of phenylacetic acid to reduce biodegradation inhibition. Experimental results (Fig. 6b) indicated that the rate of phenylacetic acid oxidation can be accelerated by visible light.

Photocatalysts are important components in traditional photobiological degradation processes. In our study, the penicillin treatment process was accomplished using light-irradiation assisted biocatalysts without other photocatalysts. Penicillin was biodegraded by enzymes from KDSPL-02. Light irradiation played an important role to accelerate the biodegradation rate of KDSPL-02. We speculated that several enzyme candidates in the oxidation process could respond to light irradiation. The traditional photocatalysis process requires a photo-oxidant. In the process of photooxidation, the substrate usually transforms to a hyper-oxidation product [37]. These hyper-oxidation products cannot be further eliminated, which may cause secondary pollution. Due to the unique light sensitivity of KDSPL-02, penicillin biodegradation activated by light was achieved. Because no additional photocatalysts were added in the combined biodegradation and photocatalysis process, the process for separation was simplified, which reduced production costs. This process is more efficient and environmentally friendly.

Further research could greatly expand the scope of application of the bacterial strain, especially in the treatment of antibiotic waste water, industrial bacterial residue, and rivers around pharmaceutical factories. In terms of the biodegradative inhibition of benzoic acid in KDSPL-02, in situ product removal has shown advantages in improving production [46, 47], Further research is warranted to determine whether these advantages are applicable to biodegradation.

The whole genome of KDSPL-02 was analysed. The PPA pathway from bacteria can degrade phenylacetic acid, and the degradation product can be introduced to the central metabolism of microorganisms for mineralization [48]. In genomic mining,. Enzymes PaaN and PaaD were both found in the genome of KDSPL-02. The genomic information will be shown in our future report. We speculated that light irradiation could promote the transcription and expression of these enzymes to further accelerate the biodegradation rate of phenylacetic acid.

## Conclusions

We identified and enriched the bacterial strain *Paracoccus* sp. KDSPL-02. The optimal conditions for penicillin biotransformation were as follows: 30 °C, pH 8.0, and an initial penicillin G concentration of 0.8–1.2 g L^− 1^. Penicillin G was completely degraded within 24 h at a concentration of 1.2 g L^− 1^. Even when the initial concentration increased above 1.6 g L^− 1^, thorough degradation was obtained via synergistic photocatalysis with whole cell KDSPL-02, which accelerated the rate-limiting transformation of penicilloic acid intermediate into phenylacetic acid and 2-(amino(carboxyl)methyl)-5,5-dimethylthiazolidine-4-carboxylic acid, respectively. The high degradation performance could be attributed to the enhanced oxidation of phenylacetic acid by photocatalysis to reduce biodegradation inhibition. The novel heterogeneous photocatalysis of whole cell KDSPL-02 could provide a new method for the treatment of residual antibiotics and provide important experimental data on penicillin behaviour in nature.

## Supplementary Information

**Additional file 1: Table S1.** Degradation capability of Penicillin G by different strain species as reported from previous literatures and this study. **Fig. S1a-c**: Structure of the identified metabolites of peak Nos. 2 (a), 3 (a), 4 (b), and 5 (c) by LC–MS analysis. Peak Nos. 2 and 3 in HPLC: Potassium 2-(carboxy(2-phenylacetamido)methyl)-5,5-dimethylthiazolidine-4-carboxylate and potassium 2-(4-carboxy-5,5-dimethylthiazolidin-2-yl)-2-(2-phenylacetamido) acetate. Peak No. 4 in HPLC: Phenylacetic acid. Peak No. 5 in HPLC: Mixture of two isomers, 2-(amino(carboxyl)methyl)-5,5-dimethylthiazolidine-4-carboxylic acid. **Fig. S2a-b**: ^1^H NMR (a) and ^13^C NMR (b) spectra of potassium 2-(carboxy(2-phenylacetamido)methyl)-5,5-dimethylthiazolidine-4-carboxylate and potassium 2-(4-carboxy-5,5-dimethylthiazolidin-2-yl)-2-(2-phenylacetamido) acetate. ^1^H NMR (500 MHz, D_2_O) δ:7.34–7.45 (m, 10H); 5.07–5.06 (d, J = 3 Hz, 1H); 5.05–5.06 (d, J = 6 Hz, 1H); 4.79–4.78 (d, J = 3 Hz, 2H), 4.25–4.24 (d, *J* = 6 Hz, 1H); 3.81–3.80 (d, *J* = 4.0 Hz, 2H); 3.72–3.70 (d, *J* = 12 Hz, 1H); 3.43–3.42 (d, *J* = 3.5 Hz, 2H); 1.57 (s, 3H); 1.51 (s, 3H); 1.23 (s,3H); 1.05 (s, 3H).^13^C NMR (125 MHz, D_2_O) δ:176.17, 175.58, 175.24, 174.93, 174.86, 174.30, 135.01, 134.50, 129.65(2C), 129.49(2C), 129.27(2C), 129.04(2C), 127.61, 127.42, 75.84, 75.28, 67.00, 66.02, 60.01, 58.61, 58.45, 55.24, 42.51, 42.46, 27.98, 27.75, 26.75, 26.33. LC–MS (m/z): 391.3[M + 1]^+^ (cald. For C_16_H_19_KN_2_O_5_S, 390.1), 353.2[M` + 1]^+^ (cald for C_16_H_20_N_2_O_5_S, 352.1). **Fig. S3a-b**: ^1^H NMR (a) and ^13^C NMR (b) spectra of phenylacetic acid. ^1^H NMR (500 MHz, D_2_O) δppm: 7.42–7.32 (m, 5H); 3.73 (s, 2H).^13^CNMR (125 MHz, D_2_O) δppm: 177.01, 134.17, 129.38(2C), 128.79(2C), 127.27, 40.54. LC–MS (*m/z*): 137.3[M + 1]^**+**^ (cald. For C_8_H_8_O_2_, 136.05). **Fig. S4a-b**: ^1^H NMR (a) and ^13^C NMR (b) spectra of 5,5-dimethylthiazolidine. ^1^H NMR (500 MHz, D_2_O) δ: 5.06–5.05 (d, J = 4.5 Hz, 1H); 4.85–4.83 (d, J = 10.0 Hz, 1H); 3.97–3.96 (d, J = 4.0 Hz, 1H); 3.64–3.62 (d, J = 10.0 Hz, 1H); 3.56 (s, 2H); 3.47 (s, 1H); 3.19 (s,1H); 1.45 (s, 3H); 1.44 (s, 3H); 1.19 (s, 3H); 1.13(s, 3H). ^13^C NMR (125 MHz, D_2_O) δ: 176.34, 174.38, 171.85, 171.20, 74.47, 74.00, 63.59, 63.29, 59.31, 58.68, 56.80, 56.39, 29.29, 26.06, 26.02, 25.94. LC–MS (*m/z*): 235.1 [M + 1]^**+**^ (cald for C_8_H_14_N_2_O_4_S, 234.07).

## Data Availability

Not applicable.
