# Supplementary material for: Assessment of the efficiency of synergistic photocatalysis on penicillin G biodegradation by whole cell Paracoccus sp
Source: J Biol Eng. 2021 Oct 27;15:25. doi: 10.1186/s13036-021-00275-4 (PMC8554860; doi:10.1186/s13036-021-00275-4)
Supplement: Supplementary file 1 — Additional file 1: Table S1. Degradation capability of Penicillin G by different strain species as reported from previous literatures and this study. Fig. S1a-c: Structure of the identified metabolites of peak Nos. 2 (a), 3 (a), 4 (b), and 5 (c) by LC–MS analysis. Peak Nos. 2 and 3 in HPLC: Potassium 2-(carboxy(2-phenylacetamido)methyl)-5,5-dimethylthiazolidine-4-carboxylate and potassium 2-(4-carboxy-5,5-dimethylthiazolidin-2-yl)-2-(2-phenylacetamido) acetate. Peak No. 4 in HPLC: Phenylacetic acid. Peak No. 5 in HPLC: Mixture of two isomers, 2-(amino(carboxyl)methyl)-5,5-dimethylthiazolidine-4-carboxylic acid. Fig. S2a-b: 1H NMR (a) and 13C NMR (b) spectra of potassium 2-(carboxy(2-phenylacetamido)methyl)-5,5-dimethylthiazolidine-4-carboxylate and potassium 2-(4-carboxy-5,5-dimethylthiazolidin-2-yl)-2-(2-phenylacetamido) acetate. 1H NMR (500 MHz, D2O) δ:7.34–7.45 (m, 10H); 5.07–5.06 (d, J = 3 Hz, 1H); 5.05–5.06 (d, J = 6 Hz, 1H); 4.79–4.78 (d, J = 3 Hz, 2H), 4.25–4.24 (d, J = 6 Hz, 1H); 3.81–3.80 (d, J = 4.0 Hz, 2H); 3.72–3.70 (d, J = 12 Hz, 1H); 3.43–3.42 (d, J = 3.5 Hz, 2H); 1.57 (s, 3H); 1.51 (s, 3H); 1.23 (s,3H); 1.05 (s, 3H).13C NMR (125 MHz, D2O) δ:176.17, 175.58, 175.24, 174.93, 174.86, 174.30, 135.01, 134.50, 129.65(2C), 129.49(2C), 129.27(2C), 129.04(2C), 127.61, 127.42, 75.84, 75.28, 67.00, 66.02, 60.01, 58.61, 58.45, 55.24, 42.51, 42.46, 27.98, 27.75, 26.75, 26.33. LC–MS (m/z): 391.3[M + 1]+ (cald. For C16H19KN2O5S, 390.1), 353.2[M` + 1]+ (cald for C16H20N2O5S, 352.1). Fig. S3a-b: 1H NMR (a) and 13C NMR (b) spectra of phenylacetic acid. 1H NMR (500 MHz, D2O) δppm: 7.42–7.32 (m, 5H); 3.73 (s, 2H).13CNMR (125 MHz, D2O) δppm: 177.01, 134.17, 129.38(2C), 128.79(2C), 127.27, 40.54. LC–MS (m/z): 137.3[M + 1]+ (cald. For C8H8O2, 136.05). Fig. S4a-b: 1H NMR (a) and 13C NMR (b) spectra of 5,5-dimethylthiazolidine. 1H NMR (500 MHz, D2O) δ: 5.06–5.05 (d, J = 4.5 Hz, 1H); 4.85–4.83 (d, J = 10.0 Hz, 1H); 3.97–3.96 (d, J = 4.0 Hz, 1H); 3.64–3.62 (d, J = 10.0 Hz, 1H); [file 13036_2021_275_MOESM1_ESM.doc]

**Supplementary Material**

**Assessment of the efficiency of synergistic photocatalysis on Penicillin G biodegradation by whole cell Paracoccus sp.**

Peng Wang^1,2,3^*, Xiaochun Wang^1^, Qinqin Cong^1^ and Jialin Lu^1^

^1^ College of Chemical & Pharmaceutical Engineering, Hebei University of Science & Technology, Shijiazhuang, 050018, China

^2^ State Key Laboratory Breeding Base-Hebei Province Key Laboratory of Molecular Chemistry for Drug, Hebei University of Science & Technology, Shijiazhuang, 050018, China

^3^ Hebei Province Pharmaceutical Chemical Engineering Technology Research Center, Shijiazhuang, 050018, China

Peng Wang: e-mail: [pwang@hebust.edu.cn](mailto:pwang@hebust.edu.cn)

*Corresponding author

College of Chemical & Pharmaceutical Engineering,

Hebei University of Science & Technology, Shijiazhuang,

050018, China

Tel.: +86-311-81668397

**Table S1 Degradation capability of Penicillin G by different strain species as reported from previous literatures and this study.**

| Species | PEN G concentration (mg/L) | Degradation rate (%) | Reference |
| --- | --- | --- | --- |
| *Klebsiella pneumoniae* Z-1 | 300 | 99 | Wang et al. (2015) |
| *Phanerochate chrysosporium* | 2000 | 61.4 | Mullai et al. (2011) |
| *Actinobacillus pleuropneumoniae* | 1 | 100 | Hathroubi et al. (2015) |
| *Achromobacter* sp. CCM 2428 | 500 | 100 | Skrob et al. (2003) |
| *Paracoccus* sp. KDSPL-02 | 1200 | 100 | This study |

**
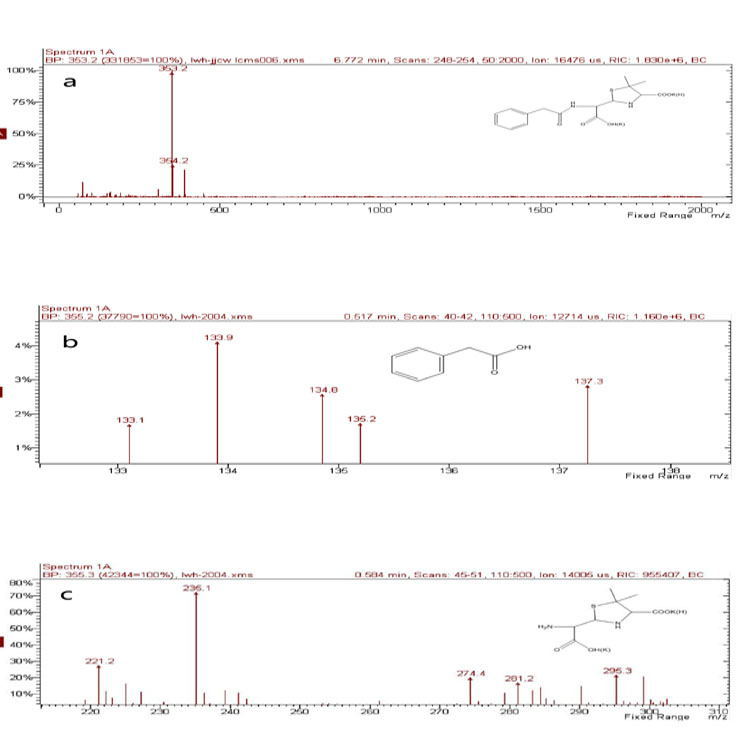
**

**Fig. S1a-c**: Structure of the identified metabolites of peak Nos. 2 (a), 3 (a), 4 (b), and 5 (c) by LC–MS analysis. Peak Nos. 2 and 3 in HPLC: Potassium 2-(carboxy(2-phenylacetamido)methyl)-5,5-dimethylthiazolidine-4-carboxylate and potassium 2-(4-carboxy-5,5-dimethylthiazolidin-2-yl)-2-(2-phenylacetamido) acetate. Peak No. 4 in HPLC: Phenylacetic acid. Peak No. 5 in HPLC: Mixture of two isomers, 2-(amino(carboxyl)methyl)-5,5-dimethylthiazolidine-4-carboxylic acid.


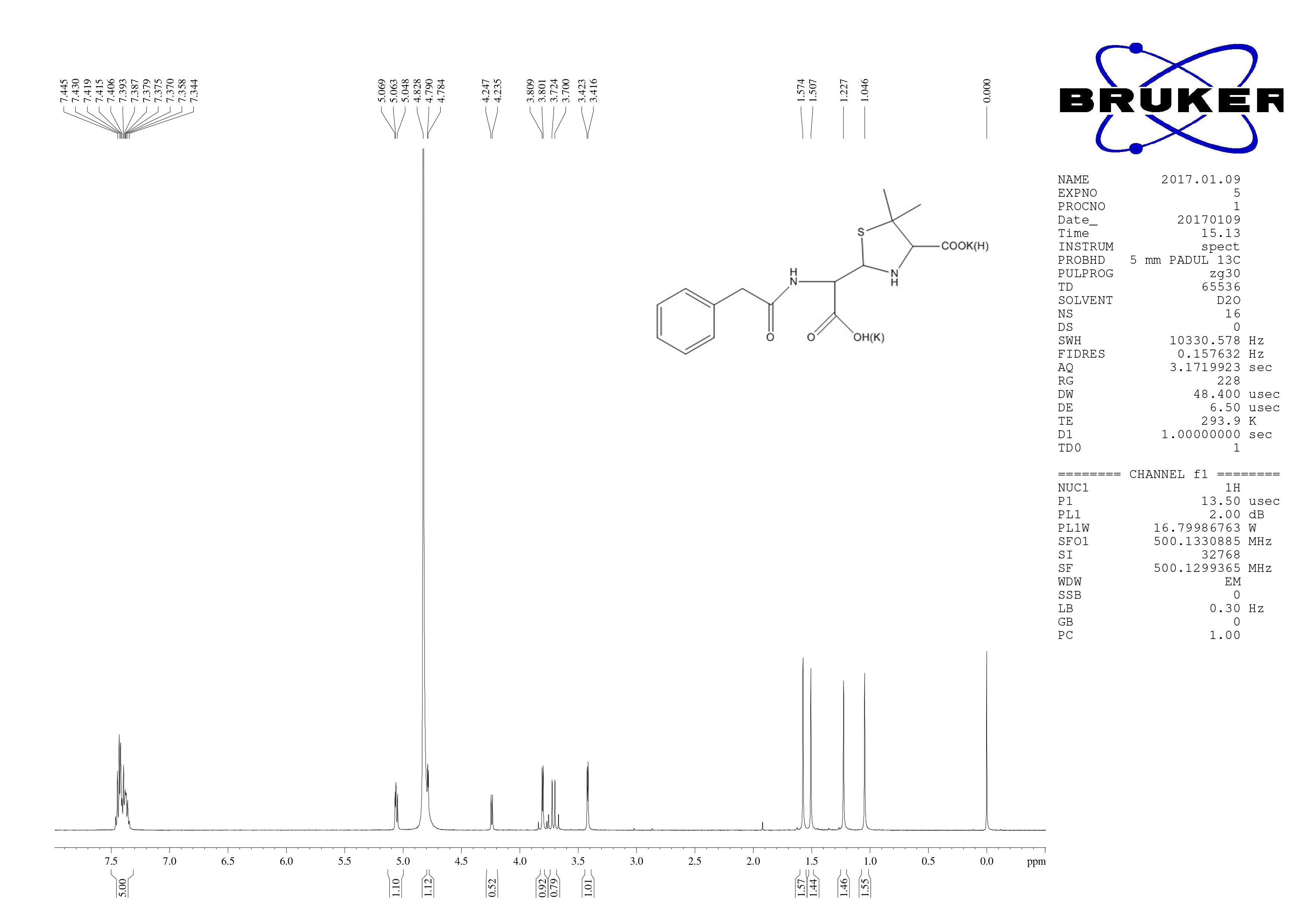


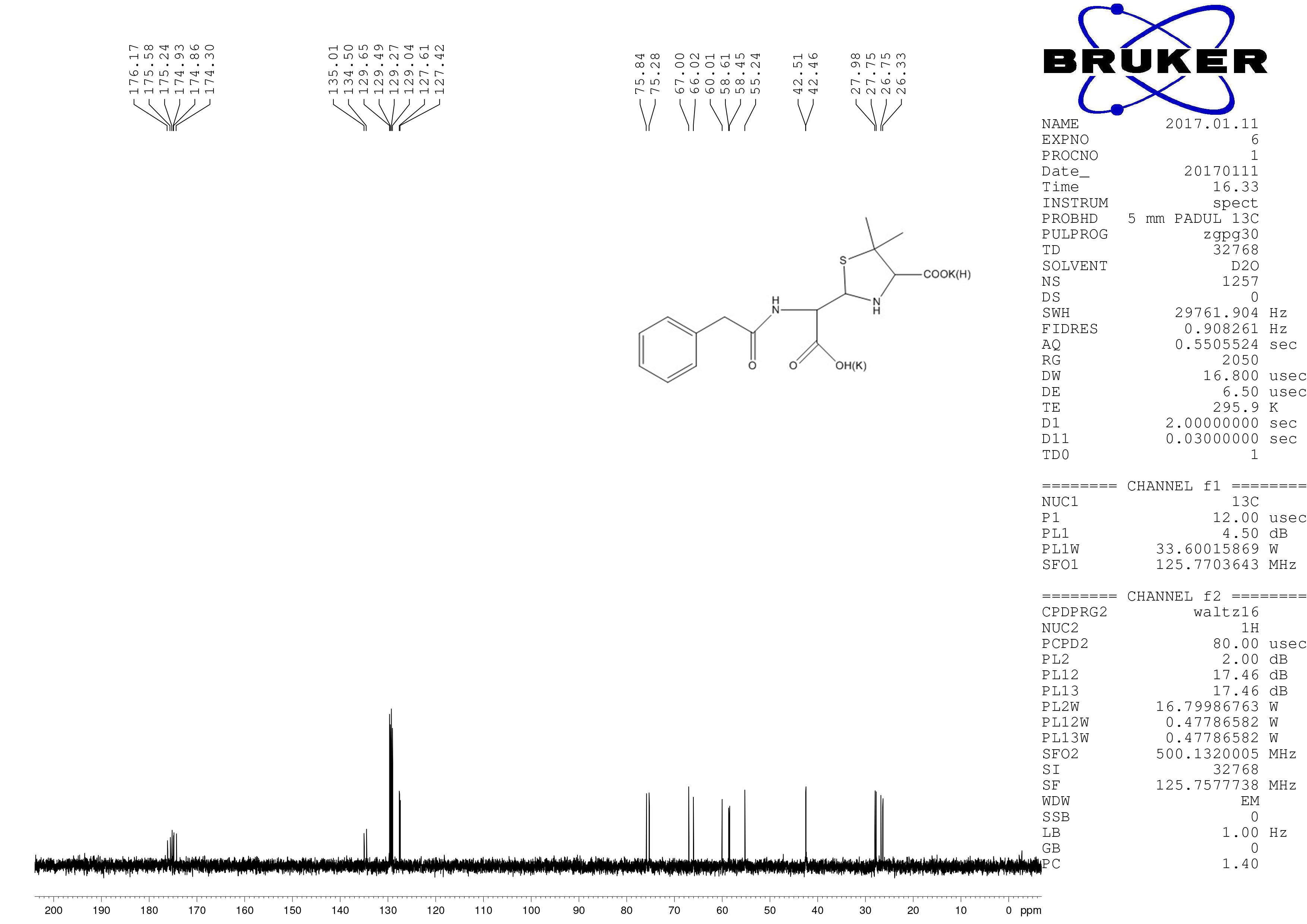


**Fig. S2a-b**: ^1^H NMR (a) and ^13^C NMR (b) spectra of potassium 2-(carboxy(2-phenylacetamido)methyl)-5,5-dimethylthiazolidine-4-carboxylate and potassium 2-(4-carboxy-5,5-dimethylthiazolidin-2-yl)-2-(2-phenylacetamido) acetate. ^1^H NMR (500 MHz, D_2_O) δ:7.34–7.45 (m, 10H); 5.07–5.06 (d, J = 3 Hz, 1H); 5.05–5.06 (d, J = 6 Hz, 1H); 4.79–4.78 (d, J=3Hz, 2H), 4.25–4.24 (d, *J* = 6 Hz, 1H); 3.81–3.80 (d, *J* = 4.0 Hz, 2H); 3.72–3.70 (d, *J* = 12 Hz, 1H); 3.43–3.42 (d, *J* = 3.5 Hz, 2H); 1.57 (s, 3H); 1.51 (s, 3H); 1.23 (s,3H); 1.05 (s, 3H).^13^C NMR (125 MHz, D_2_O) δ:176.17, 175.58, 175.24, 174.93, 174.86, 174.30, 135.01, 134.50, 129.65(2C), 129.49(2C), 129.27(2C), 129.04(2C), 127.61, 127.42, 75.84, 75.28, 67.00, 66.02, 60.01, 58.61, 58.45, 55.24, 42.51, 42.46, 27.98, 27.75, 26.75, 26.33. LC–MS (m/z): 391.3[M+1]^+^ (cald. for C_16_H_19_KN_2_O_5_S, 390.1), 353.2[M`+1]^+^ (cald for C_16_H_20_N_2_O_5_S, 352.1).


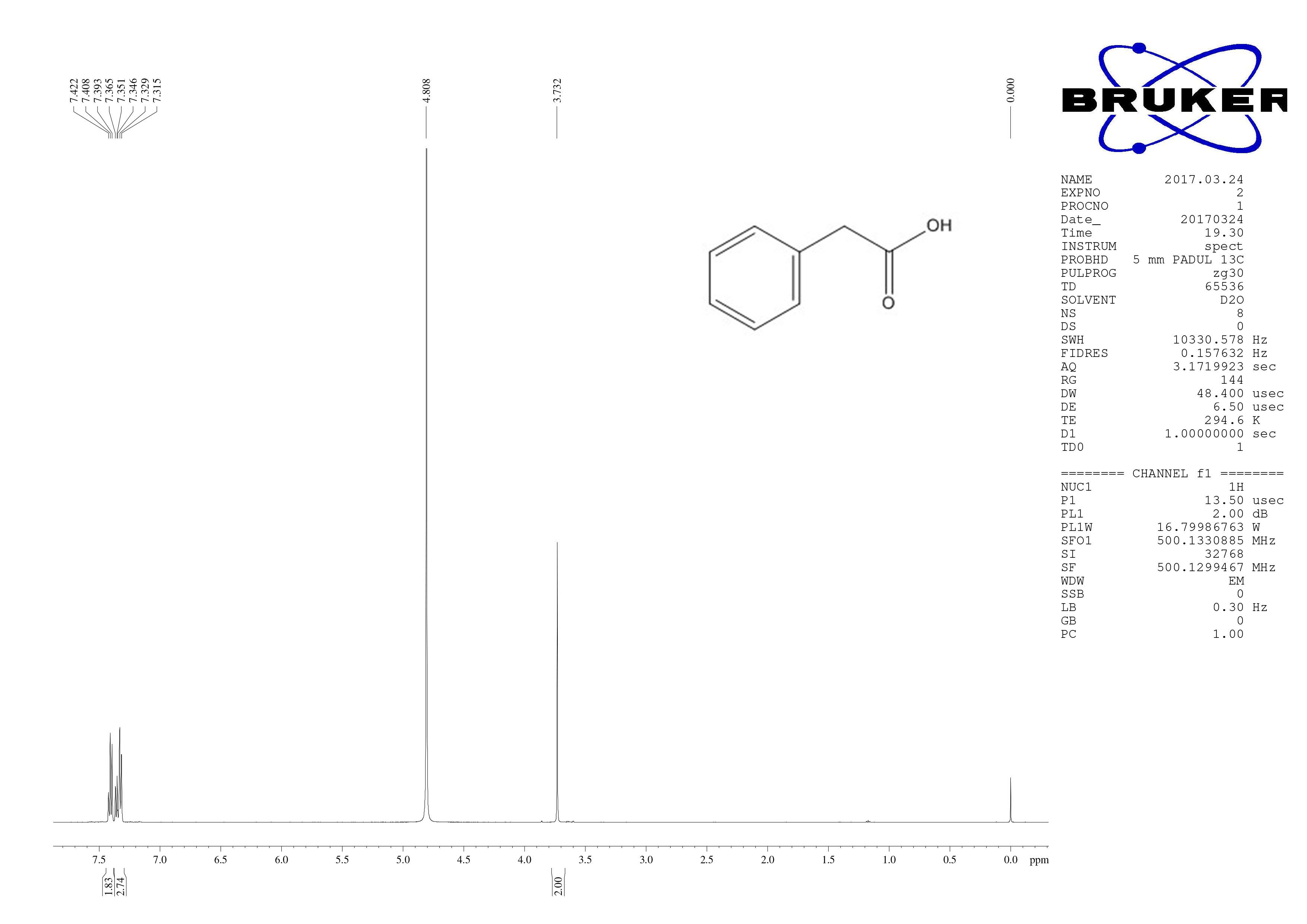


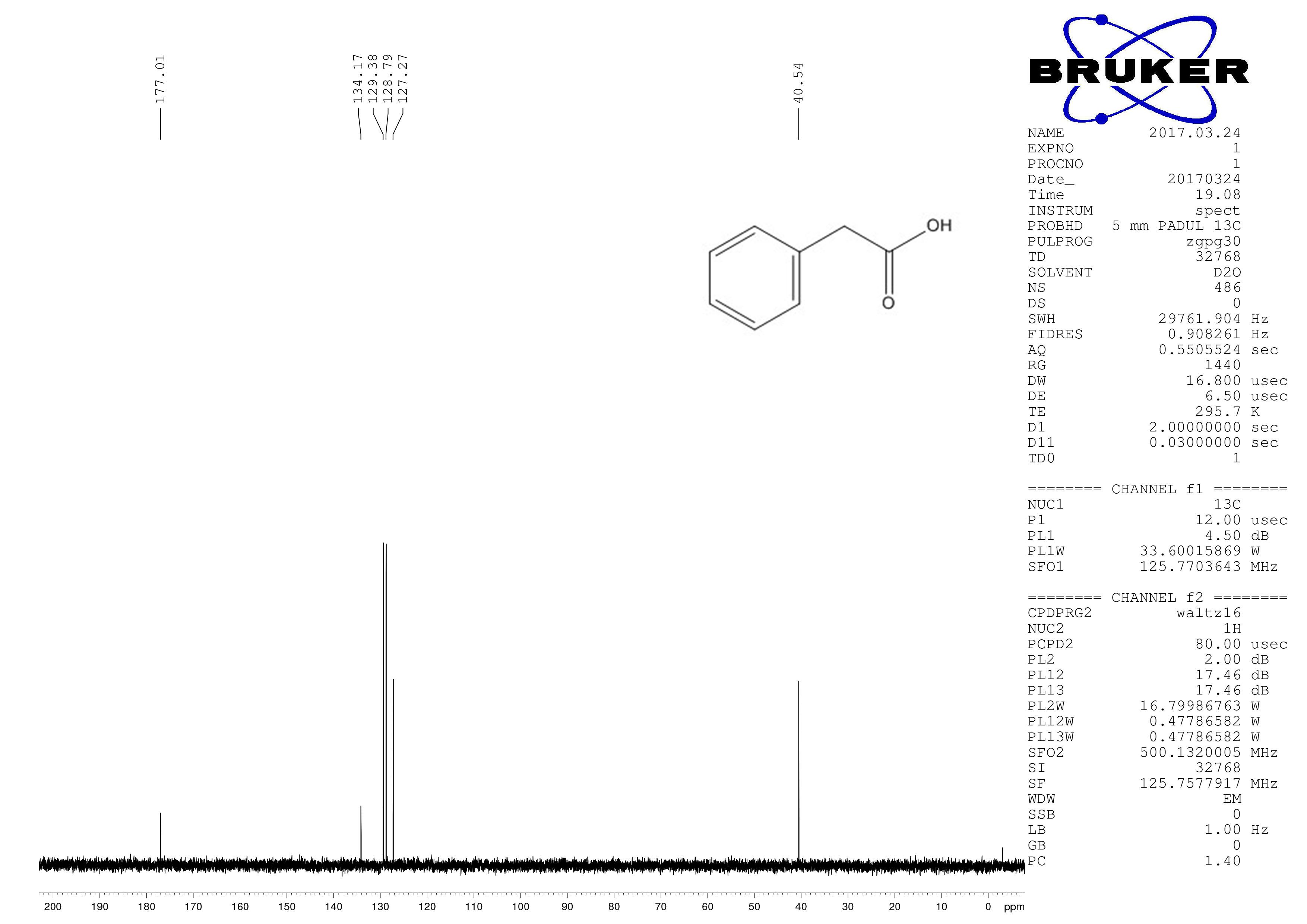


**Fig. S3a-b**: ^1^H NMR (a) and ^13^C NMR (b) spectra of phenylacetic acid. ^1^H NMR (500 MHz, D_2_O) δppm: 7.42–7.32 (m, 5H); 3.73 (s, 2H).^13^CNMR (125 MHz, D_2_O) δppm: 177.01, 134.17, 129.38(2C), 128.79(2C), 127.27, 40.54. LC–MS (*m/z*): 137.3[M+1]**^+^** (cald. for C_8_H_8_O_2_, 136.05).


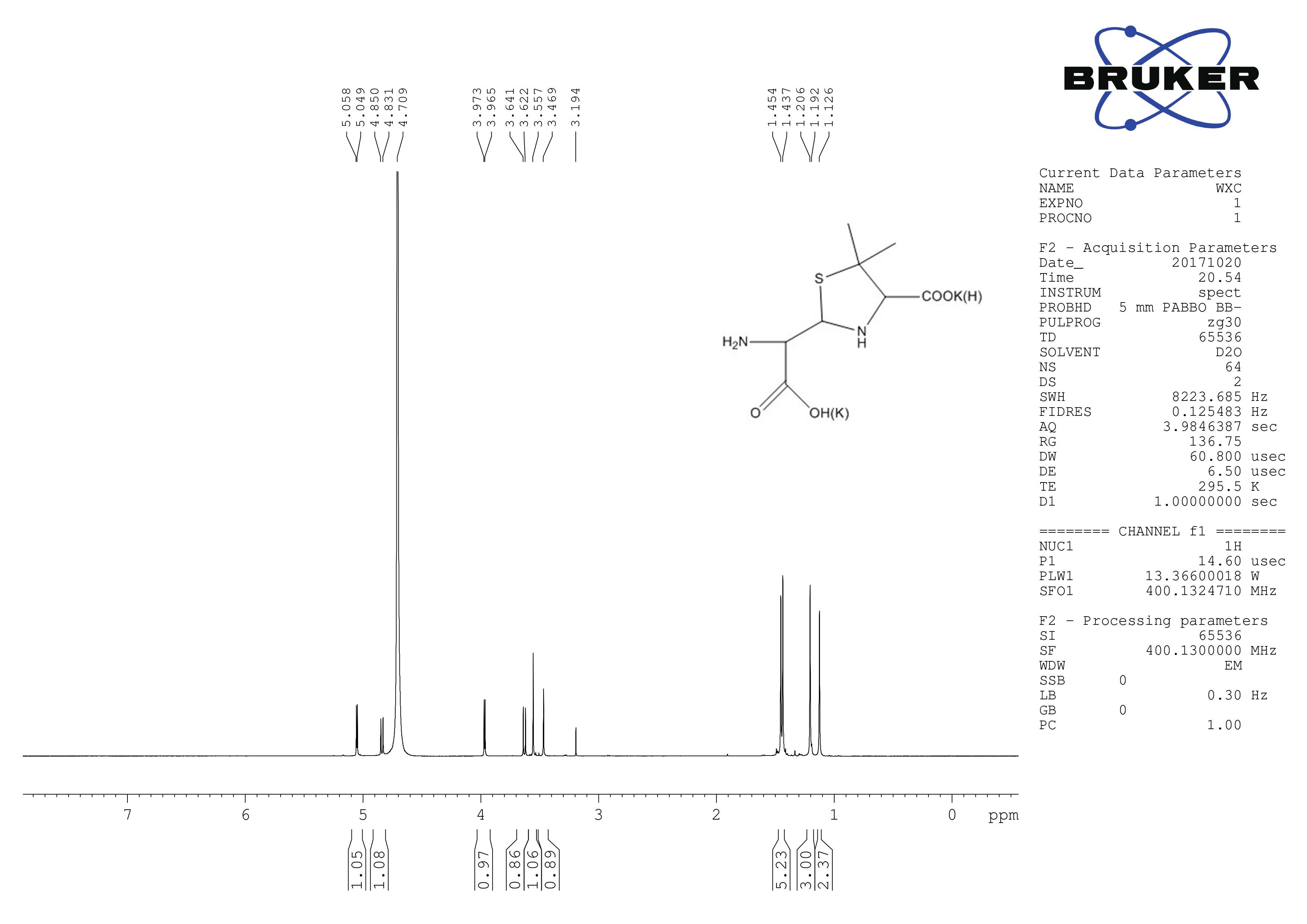


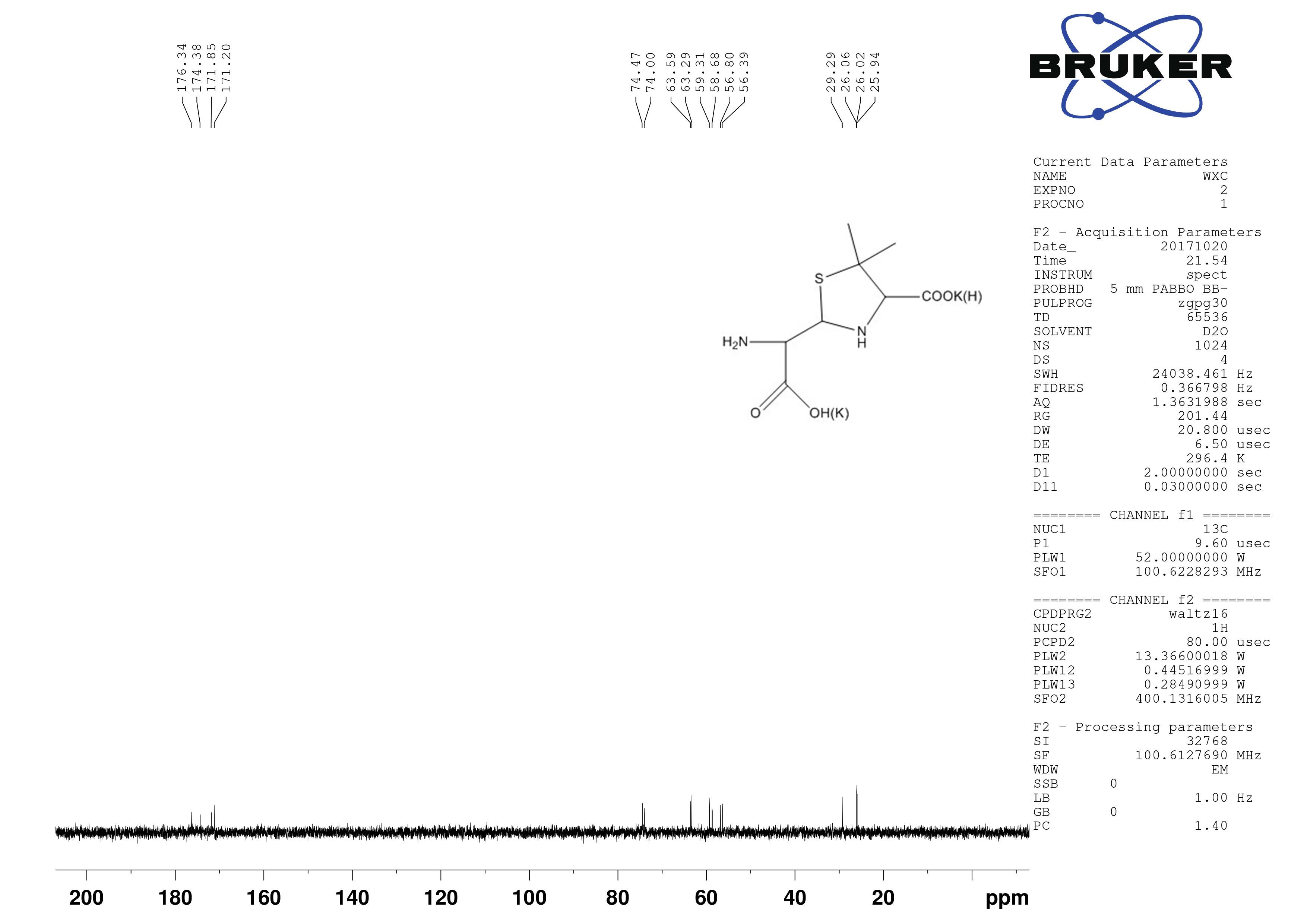


**Fig. S4a-b**: ^1^H NMR (a) and ^13^C NMR (b) spectra of 5,5-dimethylthiazolidine. ^1^H NMR (500 MHz, D_2_O) δ: 5.06–5.05 (d, J= 4.5 Hz, 1H); 4.85–4.83 (d, J=10.0 Hz, 1H); 3.97–3.96 (d, J=4.0 Hz, 1H); 3.64–3.62 (d, J=10.0 Hz, 1H); 3.56 (s, 2H); 3.47 (s, 1H); 3.19 (s,1H); 1.45 (s, 3H); 1.44 (s, 3H); 1.19 (s, 3H); 1.13(s, 3H). ^13^C NMR (125 MHz, D_2_O) δ: 176.34, 174.38, 171.85, 171.20, 74.47, 74.00, 63.59, 63.29, 59.31, 58.68, 56.80, 56.39, 29.29, 26.06, 26.02, 25.94. LC–MS (*m/z*): 235.1 [M+1]**^+^** (cald for C_8_H_14_N_2_O_4_S, 234.07).
